# Dopaminergic D1 receptor effects on commissural inputs targeting layer V pyramidal subtypes of the mouse medial prefrontal cortex

**DOI:** 10.14814/phy2.14256

**Published:** 2019-10-24

**Authors:** Jonna M. Leyrer‐Jackson, Mark P. Thomas

**Affiliations:** ^1^ School of Psychology Psychology Department – Behavioral Neuroscience Arizona State University Tempe Arizona; ^2^ School of Biological Sciences University of Northern Colorado Greeley Colorado

**Keywords:** Dopamine, Prefrontal Cortex, EPSP, Channelrhodopsin‐2

## Abstract

In humans, prefrontal cortical areas are known to support goal‐directed behaviors, mediating a variety of functions that render behavior more flexible in the face of changing environmental demands. In mice, these functions are mediated by homologous regions within medial prefrontal cortex (mPFC) and rely heavily on proper dopaminergic tone. Comprised of two major subtypes, pyramidal tract (PT) and intratelencephalic (IT), layer V pyramidal cells serve as the major outputs of the mPFC, targeting brainstem nuclei and the contralateral hemisphere, respectively. However, it remains relatively unknown how cortical inputs targeting these subtypes are integrated. We explored how layer V pyramidal cell subtypes integrate commissural inputs, which integrate information flow between the hemispheres. An optogenetic approach was used to elicit commissural fiber activation onto PT and IT cells and the effects of D1 receptor activation on elicited EPSPs were explored. We showed that commissural inputs into PT and IT cells elicit facilitating and depressing EPSP patterns, respectively. D1 receptor activation increased the initial EPSP amplitude, enhanced EPSP facilitation, and prolonged EPSP decay time constant in PT cells. In IT cells, D1 receptor activation increased commissural‐evoked initial EPSP amplitude but did not affect facilitation or EPSP shape. Furthermore, D1 receptor activation elicited burst firing in a subset of PT cells in response to commissural fiber activation. Combined, these results lend insight into the role of dopamine in promoting persistent firing and temporal integration in PT and IT cells, respectively, that in turn may contribute to working memory functions.

## Introduction

In humans, prefrontal cortical (PFC) areas are known to support goal‐directed behaviors, rendering behavior more flexible during changing environmental demands. It has been hypothesized that the function of medial regions of the PFC is to learn associations between context, events, and corresponding emotional responses, that is, action–outcome associations (Euston et al., 2012). These associations are utilized in the performance of executive functions such as working memory tasks and attentional control that are critically dependent on prefrontal circuits. These functions are mediated by homologous regions in the medial prefrontal cortex (mPFC), in mice (Heidbreder and Groenewegen, 2003; Seamans et al., 2008).

Normal prefrontal cortical function is critically dependent on dopaminergic input from the ventral tegmental area of the midbrain (Goldman‐Rakic, 1995). Dopamine, acting on D1‐like and D2‐like receptors in the PFC, has been implicated in a wide variety of prefrontal functions, including updating working memory representations (Sawaguchi and Goldman‐Rakic, 1991) and contextual representations (D'Ardenne et al., 2012), as well as rewarding appetitive behaviors (Arias‐Carrión and Pŏppel, 2007). Expression of dopamine receptor subtypes shows cellular localization within the mPFC, with D1 receptors being substantially greater than D2 receptors on pyramidal neurons (Gaspar et al., 1995; Floresco, 2013). Studies within the mPFC have revealed that dopamine, acting through D1‐type receptors, regulates cognition in a dose‐dependent manner. That is, insufficient activation of D1 receptors leads to distractibility, while excessive activation leads to perseveration; an optimal level of D1 receptor activation is thus necessary for proper working memory function (Arnsten, 1997; Zahrt et al., 1997; Williams and Castner, 2006). Despite many studies addressing the effects of dopaminergic D1‐type receptor activation on synaptic responses in the PFC, it remains unclear how dopamine's actions at D1‐type receptors lead to these dose‐dependent effects on PFC functions. To fully understand how dopamine modulates these functions requires characterization of the effects of D1 receptor activation on glutamatergic synaptic responses targeting cortical pyramidal neurons.

Of the several cortical inputs targeting the mPFC, it is known that commissural fibers from the mPFC innervate layers I through VI of the contralateral hemisphere, but have the highest density within layer V (Dembrow et al., 2015). The synaptic dynamics of these commissural inputs have been partly characterized, differing between the two subtypes of layer V cells, PT (subcortical projecting or pyramidal tract) and IT (contralateral projecting or intratelencephalic) (Molnár and Cheung, 2006; Dembrow et al., 2015). However, the modulatory effects of dopaminergic receptor activation on these synaptic dynamics are unknown. In previous studies, we demonstrated that dopamine has cell compartment and receptor subtype‐specific effects on excitatory transmission in mPFC layer V cells (Leyrer‐Jackson and Thomas, 2017; Leyrer‐Jackson and Thomas, 2018). In the current study, we used optogenetic techniques to characterize the effects of D1 receptor activation on short‐term synaptic dynamics of commissural inputs into both subtypes of layer V pyramidal neurons. We showed that D1 receptor activation has differential effects on EPSP trains in the two subtypes of layer V cells; interestingly, we also showed that D1 receptor activation in conjunction with commissural fiber activation elicited bursting behavior in a subset of PT cells.

## Materials and Methods

### Dual Injection Stereotaxic Surgeries

**Figure 1:**
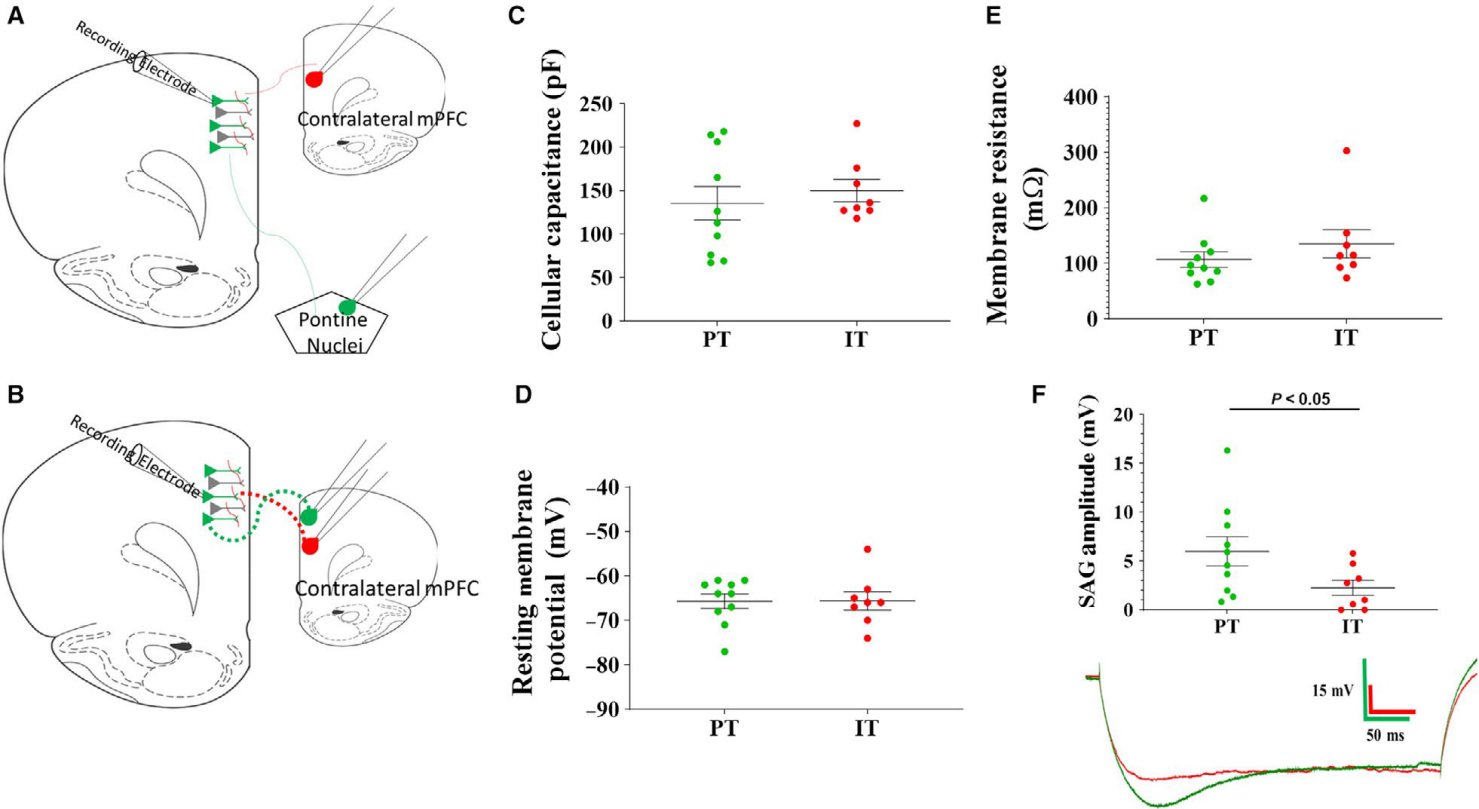
Layer V pyramidal cells of the prefrontal cortex were defined by axonal projections and intrinsic properties. Schematic representations of retrograde and channelrhodopsin‐2 labeling. AAV‐encased channelrhodopsin‐2 construct was injected into layer V of the prefrontal cortex contralateral to the recorded hemisphere (A&B). PT and IT cells were labeled with green retrobeads injected into the pontine nuclei or the contralateral PFC, respectively (PT (A); IT (B)). PT and IT cells did not differ in cellular capacitance (C), resting membrane potential (D) or membrane resistance (E). (F) PT cells showed a significantly greater SAG in response to a hyperpolarizing current injection than IT cells. Representative SAG traces are shown for PT (green) and IT (red) cells (F, bottom). Data points within C‐F represent individual cell values.

**Figure 2:**
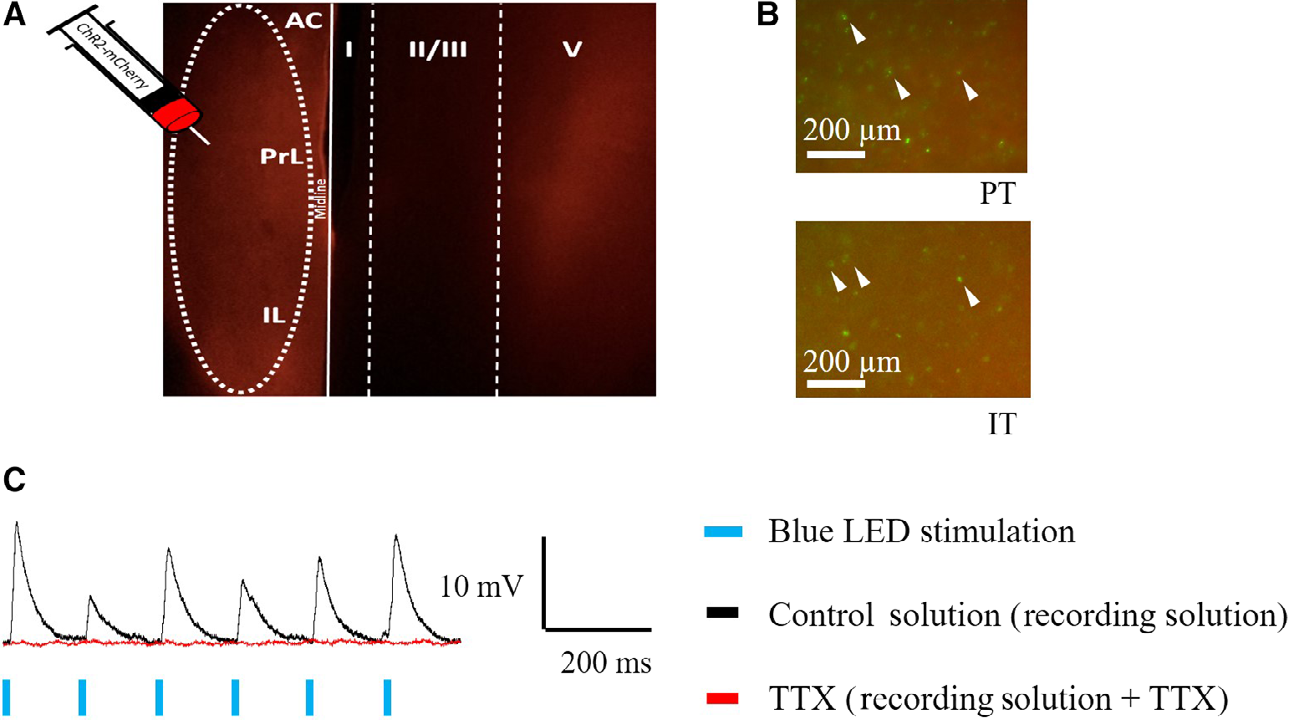
Channelrhodopsin‐2 and retrobead expression within the medial prefrontal cortex. AAV‐encased ChR2 ‐ mCherry construct was injected into layer V of the medial prefrontal cortex. Fibers containing ChR2 were visualized by mCherry and primarily targeted layer V of the contralateral hemisphere (A). (B) PT (top) and IT (bottom) layer V pyramidal cells, labeled with green retrobeads were visualized within layer V. Arrowheads depict cell bodies containing green retrobeads. (C) Optogenetically elicited EPSPs (black trace) were blocked in the presence of TTX (red trace), indicating presynaptic action potential‐evoked neurotransmitter release in response to ChR2 activation.

Stereotaxic surgeries were conducted following a UNC Institutional Animal Care and Use Committee‐approved procedure and in accordance with NIH guidelines. Young (4–6 week old) mice, of both sex, were anesthetized with a combination of isoflurane and oxygen. Once under full anesthesia, the mouse was transferred to a stereotaxic frame (Stoelting 51500U, Ultra‐Precise, Wood Dale, IL, USA), and a nose cone was placed for continuous administration of anesthesia during the surgery. Regardless of injection paradigm, two burr holes were made in the skull to inject retrobeads (Lumafluor, Naples, FL) and an adeno‐associated virus (AAV)‐encased channelrhodopsin‐2 (ChR‐2) construct tagged with mCherry (AAV9.CAG.hChR2[H134R]‐mCherry.WPRE.SV40; 1 x 10^13^ vg/mL titer; Penn Vector Core, Philadelphia, PA). For one set of experiments, PT cells and commissural fibers were labeled, where the AAV‐ChR2‐mCherry containing vector and 1X green retrobeads were injected at the coordinates, ML: +0.35 mm, DV: −2.3 to −1.3 mm, and RC:+1.8 mm and ML: −1.1, DV: −5.2 mm and RC: −3.5 mm from bregma, respectively. For experiments labeling IT cells and commissural fibers, green retrobeads and AAV‐ChR2‐mCherry were both injected at the coordinates of ML: +0.35mm, DV: −2.3 to −1.3 mm, and RC:+1.8 mm. Schematic representations of injections labeling PT or IT cells with retrobeads and commissural fibers with ChR2 are shown in Figures 1A and 2A, respectively. Coordinates for both experiments were determined based on previous literature (Gee et al., 2012; Lee et al., 2014) as well as the Paxinos Brain Atlas. Lumafluor 1X green retrobeads and the channelrhodopsin‐2 containing vector were injected with a 1 µL neuros syringe (Hamilton Company, Reno, NV, USA) at the coordinates above at volumes of 600–900 nL, each. Following injection, the incision site was sutured with 4–6 stitches (Roboz RS‐7985‐12 needles; Roboz SUT‐15‐2 sutures). Mice were then subcutaneously injected with Rimadyl (Carprofen; Pfizer Pharmaceuticals, Brooklyn, NY, USA). Mice were housed for 3–10 weeks postinjection before tissue slices were made.

### Tissue preparation

Tissue slices were prepared from 7–16 week old dual‐injected mice (C57 BL/6 strain, UNC breeding colony). Animals were anesthetized with carbon dioxide and rapidly decapitated following procedures outlined in a UNC Institutional Animal Care and Use Committee‐approved protocol in accordance with NIH guidelines. Brains were rapidly removed and immersed in ice‐cold carbogen (95% O_2_/ 5% CO_2_) saturated sucrose‐enriched artificial cerebrospinal fluid (cutting aCSF) containing (in mmol/L) sucrose, 206; NaHCO_3_, 25; dextrose, 10; KCl, 3.3; NaH_2_PO_4_, 1.23; CaCl_2_, 1.0; MgCl_2_, 4.0, osmolarity adjusted to 295 ± 5 mOsm, and pH adjusted to 7.40 ± 0.03. The brains were then transferred to the cutting chamber of a vibrating tissue slicer (OTS500, Electron Microscopy Sciences, Hatfield, PA) and coronal slices of the mPFC were prepared in ice‐cold cutting aCSF. Slices were cut 300 µm thick and were taken from approximately 200µm to 1400µmi caudal to the frontal pole. Slices were then placed in a holding chamber filled with recording aCSF solution containing (in mmol/L) NaCl, 120; NaHCO_3_, 25; KCl, 3.3; NaH_2_PO_4_, 1.23; CaCl_2_, 0.9; MgCl_2_, 2.0; dextrose, 10, osmolarity adjusted to 295 ± 5 mOsm and pH adjusted to 7.40 ± 0.03. The holding chamber aCSF was continuously bubbled with carbogen and incubated at 34°C for 45 min and then allowed to cool to room temperature before slice recording. Prior to experiments, slices were transferred to a recording chamber where they were perfused continuously at a flow rate of 1–2 mls/min with filtered, carbogen‐saturated recording aCSF solution. Throughout recordings, the recording chamber was held at 32 ± 1°C with a temperature controller equipped with a chamber heater and an in‐line heater (TC‐344B, Warner Instruments, Hamden CT).

### Electrophysiology

Retrobead‐labeled, PT and IT, layer V pyramidal neurons of the infralimbic, prelimbic, and anterior cingulate cortices were visually identified using infrared DIC microscopy at 40x objective magnification with an Olympus BX51WI microscope (Tokyo, Japan). Fluorescence was visualized using light emitted from an X‐Cite LED (Excelitas, Waltham, MA, USA). Whole cell current clamp recordings were made from the soma of fluorescently labeled layer V pyramidal neurons after establishing a Giga‐ohm seal (resistance range: 1–10 Gohm). Only cells that exhibited a thin (i.e. action potential half‐width of less than 2 msec), overshooting action potential, as well as continuous spiking throughout a depolarizing current injection were used in this study. Access resistance (R_A_) was compensated throughout experiments, and cells were excluded from analysis if uncompensated R_A_ exceeded 20MΩ. Liquid junction potentials (estimated at approximately −6 mV for K^+^ gluconate internal solution) were not compensated in adjusting Vm for synaptic recordings. Amplifier bridge balance was utilized and monitored throughout current injections. Recording pipettes (4–6 MΩ tip resistance), produced from thin‐wall glass capillary tubes (1.5 µm OD, 1.12 µm ID, World Precision Instruments, Sarasota, FL), were filled with an intracellular solution containing (in mM): potassium gluconate, 135; KCl, 10; EGTA, 1.0; HEPES, 10; MgATP, 2; TrisGTP, 0.38, osmolarity adjusted to 285 ± 5 mOsm and pH adjusted to 7.30 ± 0.01.

### Experimental protocols

While recording from PT or IT layer V pyramidal cells (labeled with fluorescent retrobeads), a blue LED was used to activate commissural fibers expressing channelrhodopsin‐2 (ChR2). A schematic representation is shown in Figure 1A and B for PT and IT cell experiments, respectively. The presence of green retrobead‐labeled PT and IT cells could be observed surrounded by ChR2‐labeled commissural fibers containing an mCherry tag. A “light intensity/ evoked response” curve was established, where the light intensity was adjusted to evoke an EPSP amplitude midrange between threshold and maximum (i.e. saturated EPSP amplitude) ranging between 4 and 12 mV. These evoked amplitudes were chosen to avoid cell spiking during the pulse trains. EPSPs were evoked at 1 Hz (to measure EPSP rise time and decay time constant) and with 8‐pulse stimulus train at 10 Hz, with a 10 sec intertrain interval. This protocol was repeated five times and the five responses were averaged. For all experiments, cells were manually held at −75 mV throughout the duration of experiment. Responses were evoked in control aCSF, immediately following a 5‐min application of the D1 agonist, SKF38393 (10 µmol/L), and immediately following a 5‐min application of the D1 antagonist, SCH23390 (10 µmol/L). All recordings were conducted in the presence of the GABA_A_ antagonist, gabazine (10 µmol/L), to isolate glutamatergic responses. Rise time was measured as the time between baseline (immediately prior to initial EPSP deflection) and peak amplitude, and decay time constant was calculated using a fitted exponential of the decay period of individual EPSPs.

### Statistical analyses

All values are presented as mean ± SEM (standard error of the mean). We performed an ANOVA on all experimental data except for comparisons between cellular properties of PT and IT cells (i.e. resting membrane potential, membrane capacitance, ‘sag’ amplitude etc.), where a Student’s t‐test was utilized. A two‐way repeated measures ANOVA was used to analyze the effects of drug and frequency of stimulation. The statistical model also included mouse and slice as random variables. For all analyses, PT and IT cells were compared separately. The responses were digitized at 10kHz and saved on disk using a Digidata 1322A interface (Axon instruments) and pCLAMP version 8.1 software (Clampex program, Axon Instruments). Data were analyzed off‐line in Clampfit (Axon Instruments).

### Drugs

The dopamine D1/D5 receptor agonist, SKF38393, the dopamine D1/D5 antagonist, SCH23390, and the GABA_a_ receptor antagonist, gabazine, were purchased from Tocris Biosciences (Bristol, UK). The sodium channel blocker, tetrodotoxin (TTX), was purchased from Ascent Scientific (now Abcam; Cambridge, MA). SKF38393, SCH23390, and gabazine were all diluted into stock aliquots of 10mmol/L. Tetrodotoxin was diluted into stock aliquots of 20mmol/L. Drug stocks were stored at −80°C and were diluted to working concentrations. Any drugs not used within 3 days of thawing were discarded.

## Results

### Electrophysiological properties differ between PT and IT layer V pyramidal cells

A total of nine animals were used in the study and a total of 17 cells were recorded (PT: *n* = 9; IT: *n* = 8). As demonstrated in previous studies (Dembrow et al., 2010; Gee et al., 2012; Spindle and Thomas, 2014; Leyrer‐Jackson and Thomas, 2017), layer V pyramidal cell subtypes identified by retrobead labeling displayed differences in their electrophysiological properties. Although the two cell types differed in some characteristics, they did not differ in membrane capacitance (PT: 135.2 ± 19.3 pF; IT: 149.9 ± 12.9 pF; *P* > 0.05), resting membrane potential (PT: −65.7 ± 1.6 mV; IT: −65.6 ± 2.0 mV; *P* > 0.05), or membrane resistance (PT: 107.2 ± 14.2 Mohm; IT: 135.6 ± 25.5 Mohm; *P* > 0.05) (Fig. 1C–E). PT cells produced a prominent depolarizing “sag” in response to a 150 pA hyperpolarizing current compared to IT cells (6.0 ± 1.5 mV sag for PT; 2.3 ± 0.8 mV sag for IT; *P* < 0.05; Fig. 1F) indicating the strong presence of the hyperpolarization‐activated cation current in PT pyramidal cells. PT cells also typically fired initial doublets in response to depolarizing current injections, which was never observed in IT cells. Both of these criteria have been used in previous studies to identify layer V subtypes (Dembrow et al., 2010; Gee et al., 2012; Lee et al., 2014; Spindle and Thomas, 2014).

### Properties of optogenetically evoked excitatory postsynaptic potentials

PT and IT cells were identified with green retrobeads, and channelrhodopsin‐2 expressing fibers were verified by mCherry expression. Representative images of ChR2 and retrobead expression are shown in Figure 2A and B. Fibers containing channelrhodopsin‐2 had kinetics fast enough to be activated at frequencies ranging from 1 Hz to 10 Hz. To ensure that the EPSPs were due to presynaptic action potential‐evoked neurotransmitter release, tetrodotoxin (TTX; 20 µmol/L) was used to block sodium channels, and therefore action potentials evoked in presynaptic axons. The blockade of action potentials inhibits neurotransmitter release from the presynaptic terminals and in turn, postsynaptic responses. When the tissue was bathed in TTX, EPSPs were eliminated (Figure 2C), indicating that the optogenetically elicited EPSPs were due to action potential‐evoked presynaptic neurotransmitter release.

### Characteristics of excitatory postsynaptic potentials evoked by commissural afferents in PT and IT pyramidal cells

**Figure 3:**
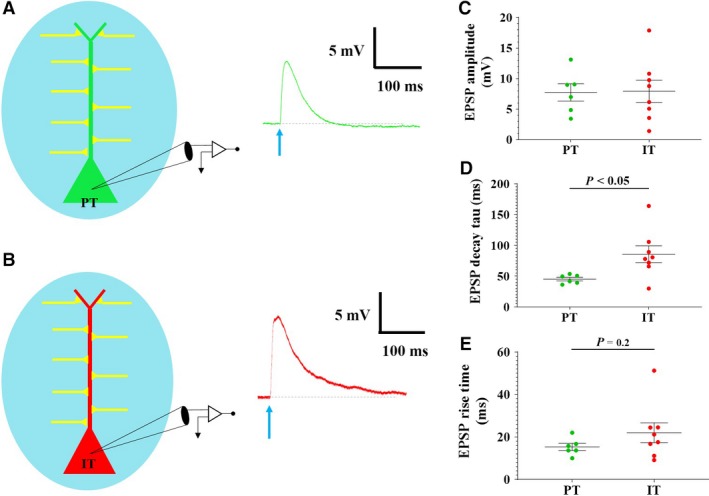
Characteristics of PT and IT EPSPs elicited by optogenetic activation of commissural fibers. (A) A schematic representation of PT recorded cell and optogenetic stimulation (left) and representative elicited EPSP (green trace; right). Blue arrow represents LED stimulation. (B) A schematic representation of IT recorded cell and optogenetic stimulation (left) and representative elicited EPSP (red trace; right). Blue arrow represents LED stimulation. (C) Elicited EPSP amplitude did not differ between PT and IT cells (*P* > 0.05; *n* = 6/8, respectively). (D) The EPSP decay time constant recorded from IT cells was significantly longer than observed in PT cells (*P* < 0.05; *n* = 6/8, respectively). (E) Although not significant, IT elicited EPSPs showed a longer rise time than observed in PT cells (*P* = 0.2; *n* = 6/8, respectively). Data points in C‐E represent individual cells.

Representative EPSP traces for a PT and IT cell, evoked at 1Hz, are shown in Figure 3A and B, respectively. EPSPs elicited in PT cells by activation of commissural inputs show an average amplitude of 7.7 ± 1.4 mV, rise time of 15.3 ± 1.7 msec and decay time constant of 45.2 ± 2.9 msec (*n* = 6). EPSPs elicited in IT cells by activation of commissural inputs show an average amplitude of 7.9 ± 1.8 mV, rise time of 22.0 ± 4.6 msec,and decay time constant of 85.7 ± 13.6 msec (*n* = 8). EPSPs elicited in IT cells had a significantly longer decay time constant than observed in PT cells (PT: 45.2 ± 2.9 msec, *n* = 6; IT: 85.7 ± 13.6 msec, *n* = 8; *P* < 0.05), however, rise times were not significantly different between the two cell types (*P* = 0.2). These data are shown in Figure 3C–E.

**Figure 4:**
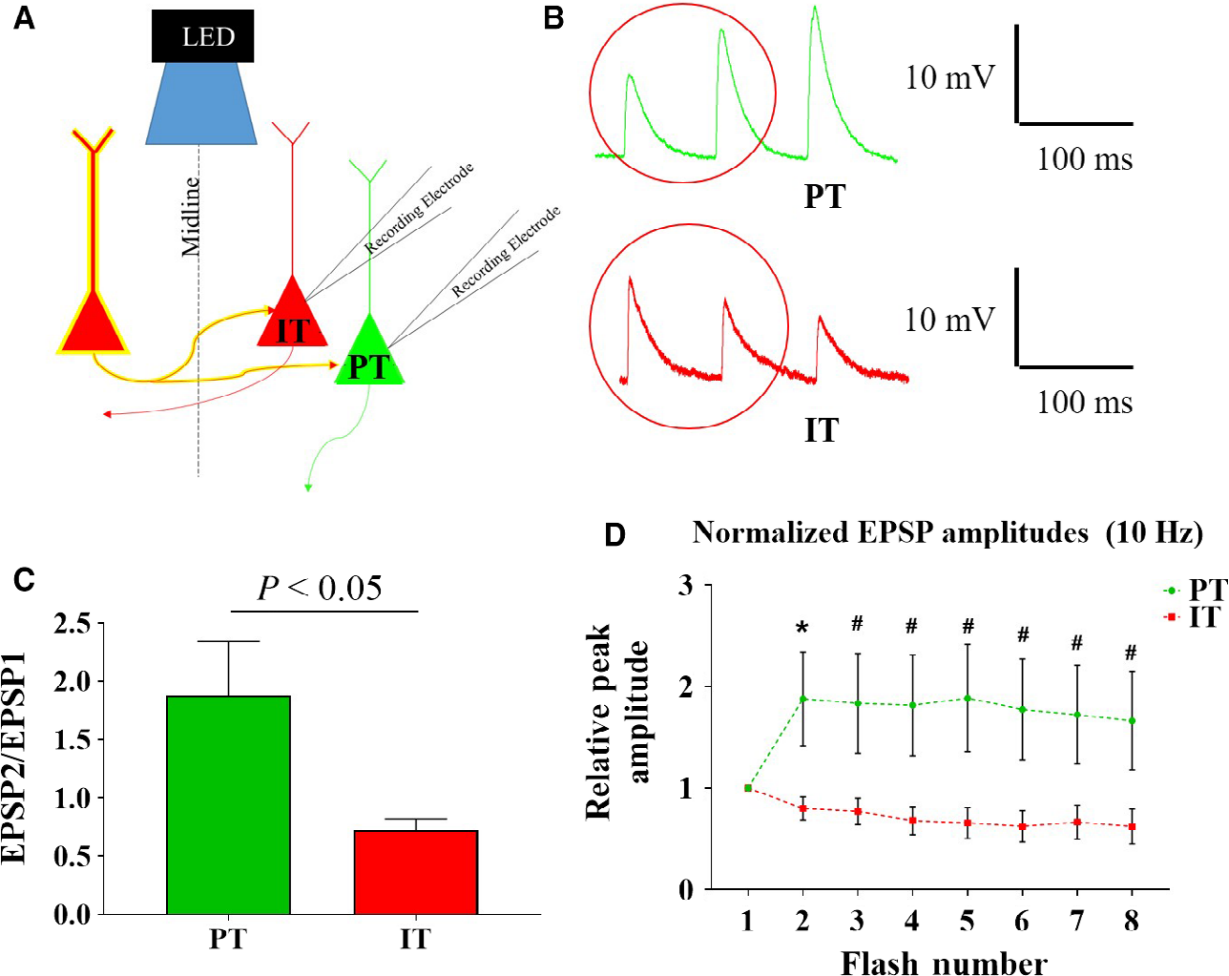
PT and IT cells display facilitating and depressing EPSP train dynamics when elicited by optogenetic activation of commissural fibers, respectively. (A) Schematic representation of recordings and optogenetic stimulation. (B) Representative traces of EPSPs elicited by commissural inputs into a PT pyramidal cell, which elicits EPSP facilitation (EPSP2> EPSP1; top; green), and onto an IT pyramidal cell, which elicits EPSP depression (EPSP2 < EPSP1; bottom; red). (C) The ratio of EPSP2/EPSP1 was significantly greater in PT than IT cells (*P* < 0.05; *n* = 9/7, respectively). (D) Normalized EPSP amplitudes during trains of light flashes evoked at 10Hz in PT (green) and IT (red) cells (*n* = 9/7, respectively). (*P < 0.05; #*P* < 0.1).

When commissural inputs into PT pyramidal cells were optogenetically activated, facilitation (EPSP2 > EPSP1) was observed in all cells (Fig. 4B, top). However, when commissural inputs into IT cells were optogenetically activated, depression (EPSP2 < EPSP1) was observed in all cells (Fig. 4B, bottom). PT and IT pyramidal cells showed an average EPSP2/EPSP1 ratio of 1.9 ± 0.5 and 0.7 ± 0.1, respectively, which was significantly different (*P* < 0.05; Fig. 4C; PT: *n* = 6; IT: *n* = 8). In PT cells, the normalized EPSP amplitude was heightened over the 8‐pulse train evoked at 10Hz, compared to IT cells (Fig. 4D). This phenomenon has also been previously reported by Lee et al. (2014).

### Effects of D1 receptor activation on evoked EPSPs in PT cells

**Figure 5:**
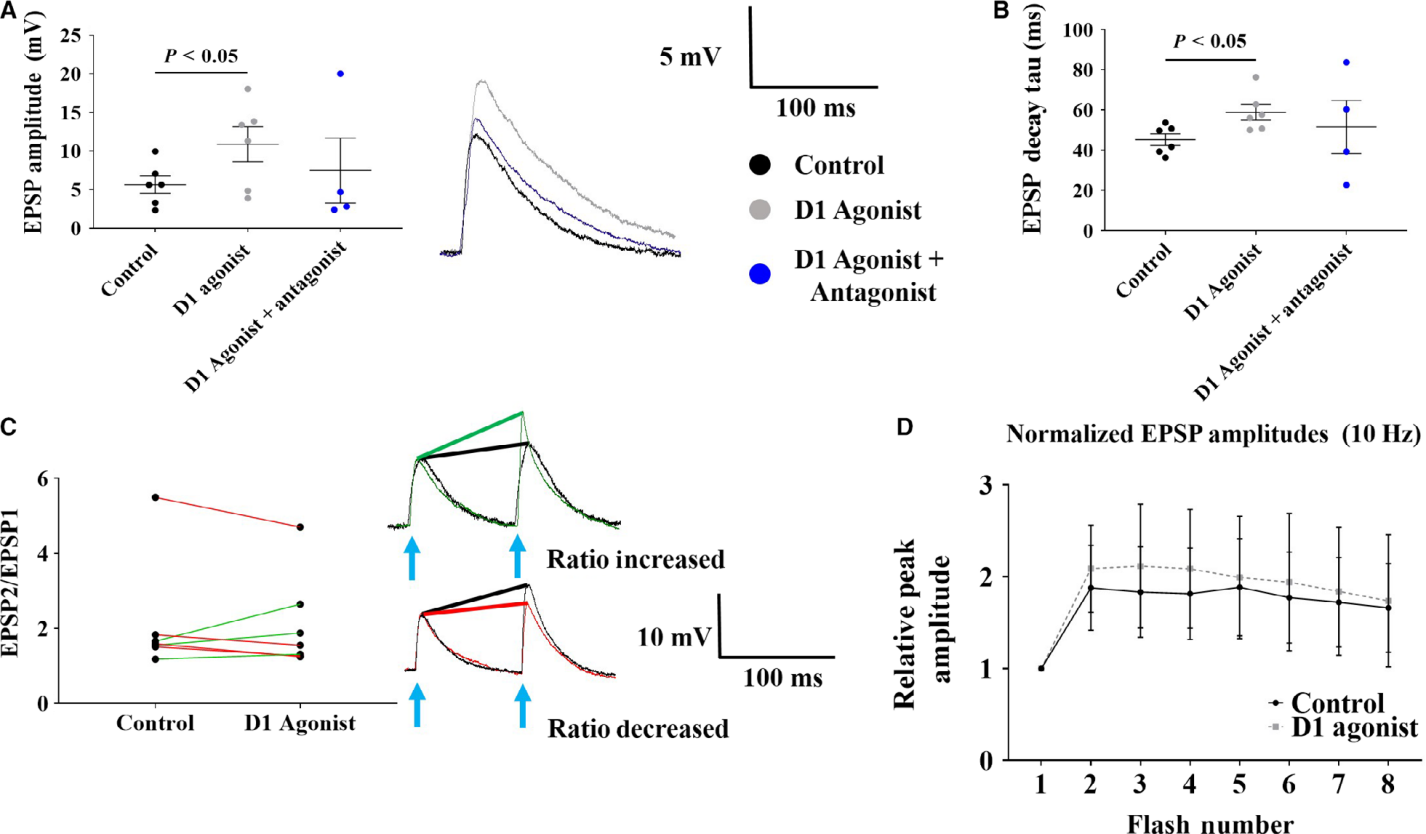
Effects of D1 receptor activation on PT EPSPs elicited by commissural fiber activation. (A) EPSP amplitude was significantly enhanced in the presence of the D1 agonist, SKF38393 (control: black (*n* = 6); D1 agonist: gray (*n* = 6)), which was partially blocked in the presence of the D1 antagonist (blue (*n* = 4)). A representative trace demonstrating this effect is shown (right). (B) D1 receptor activation significantly increased EPSP decay constant (*P* < 0.05; control: black; D1 agonist: gray). This effect was blocked in the presence of the D1 receptor antagonist (blue). (C) Facilitation at 10Hz was significantly enhanced in a subset of PT cells (green lines). Only data for cells that did not elicit bursting is shown. Cells in which a decrease in the EPSP2/1 ratio is shown in red. Of the cells that did not elicit bursting, the D1 agonist increased the EPSP2/EPSP1 in three cells and decreased the ratio in four cells. A representative trace demonstrating both of these effects is shown (right; ratio increased: top; ratio decreased; bottom). (D) Normalized EPSP amplitudes during 8‐pulse trains of light flashes in control (black) and with the D1 agonist (gray). Data points in A–C represent individual cells.

D1 receptor activation with SKF38393 had three effects on EPSPs evoked in PT cells. First, D1 receptor activation significantly increased the amplitude of EPSPs evoked at 1Hz (5.7 ± 1.1 mV in control; 10.9 ± 2.2 mV in SKF38393; *P* < 0.05; *n* = 6; Fig. 5A). This effect was partially blocked by a 5‐min application of the D1 antagonist SCH23390 (7.5 ± 4.2 mV; *P* > 0.05; *n* = 4; Fig. 5A). Second, D1 receptor activation had no significant effect on the rise time of EPSPs (15.3 ± 1.6 msec in control vs 14.9 ± 2.1 msec in SKF38393; *P* = 0.9; *n* = 6; data not shown); but significantly increased the EPSP decay time constant (45.2 ± 2.9 msec in control vs. 58.9 ± 4.0 msec in SKF38393; *P* < 0.05; *n* = 6; Fig. 5B). However, the effects on decay time constant were not blocked in the presence of the D1 agonist (51.5 ± 13.2 msec in SCH23390; *P* = 0.86; *n* = 4; Fig. 5B). Third, at 10Hz more than half of the cells recorded from (5/9) showed enhanced facilitation in the presence of the D1 agonist (i.e. the EPSP2/EPSP1 ratio was greater in the presence of the D1 agonist). Of these cells (excluding those that exhibited bursting as discussed below), facilitation was increased from 1.5 ± 0.1 in control to 1.9 ± 0.4 in the presence of the D1 agonist (Fig. 5C, green). In the remaining four cells, D1 receptor activation caused a decrease in EPSP2/EPSP1 (Fig. 5C, red traces). The effect of enhancing EPSP2/EPSP1 by D1 receptor activation was partially blocked in the presence of the D1 antagonist (1.2 ± 0.4; *P* = 0.2). Representative traces demonstrating changes in EPSP2/EPSP1 are shown in Figure 5C. Lastly, D1 receptor activation had no effect on the normalized EPSP amplitude across the 8‐pulse train at 10 Hz, compared to control (*P* > 0.05; Fig. 5D).

### Effects of D1 receptor activation on evoked EPSPs in IT cells

**Figure 6:**
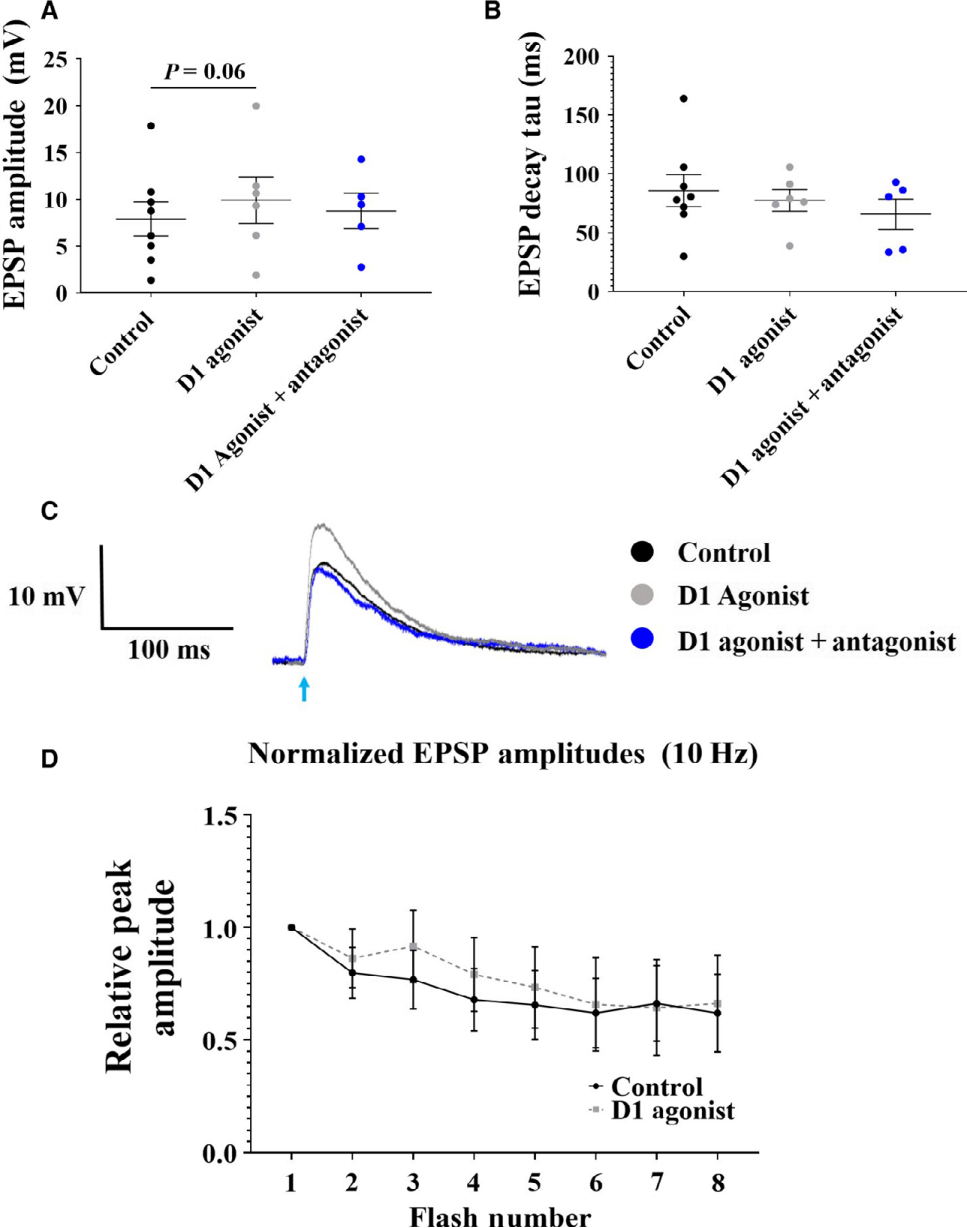
Effects of D1 receptor activation on IT EPSPs elicited by commissural fiber activation. (A) EPSP amplitude was enhanced in the presence of the D1 agonist, SKF38393 (control: black (*n* = 8); D1 agonist: gray; *P* = 0.06 (*n* = 6)), which was blocked in the presence of the D1 antagonist SCH23390 (blue; n = 5). This effect can be observed in the trace displayed in (C). (B) D1 receptor activation did not alter EPSP decay constant (control: black; D1 agonist: gray) compared to control, which was also unchanged in the presence of the D1 antagonist (blue). (C) A representative trace demonstrating this effect is shown. (D) Normalized EPSP amplitudes during 8‐pulse trains of light flashes in control (black) and with the D1 agonist (gray). Data points in A and B represent individual cells.

D1 receptor activation with SKF38393 also altered EPSPs evoked in IT cells, increasing the amplitude of EPSPs evoked at 1Hz (7.9 ± 1.8 mV in control; 9.9 ± 2.5 mV in SKF38393; *P* = 0.06; *n* = 8/6, respectively; Fig. 6A), similar to that observed in PT cells. This effect was blocked in the presence of the D1 antagonist SCH23390 (8.8 ± 1.9 mV; *P* = 0.84; *n* = 5; Fig. 6A). However, D1 receptor activation had no effect on the rise time of EPSPs (22.0 ± 4.6 msec in control vs. 25.2 ± 5.3 msec in SKF38393; *P* > 0.05; *n* = 8/6, respectively; data not shown) or EPSP decay time constant (85.7 ± 13.6 msec in control vs. 77.6 ± 9.1 msec in SKF38393; *P* > 0.05; *n* = 8/6, respectively; Fig. 6B). Decay time constant was also unaltered in the presence of the D1 antagonist (65.9 ± 12.9 msec; *P* > 0.05; *n* = 5; Fig. 6B). A representative trace of these effects is shown in Figure 6C. In contrast to PT cells, D1 receptor activation had no effect on the ratio of EPSP2/EPSP1 in IT cells at 10Hz (*P* > 0.05; data not shown). Lastly, D1 receptor activation had no effect on normalized EPSP amplitudes across the 8‐pulse train evoked at 10 Hz, compared to control (*P* > 0.05; Fig. 6D).

### D1 receptor activation elicited bursting in a subset of PT cells

**Figure 7:**
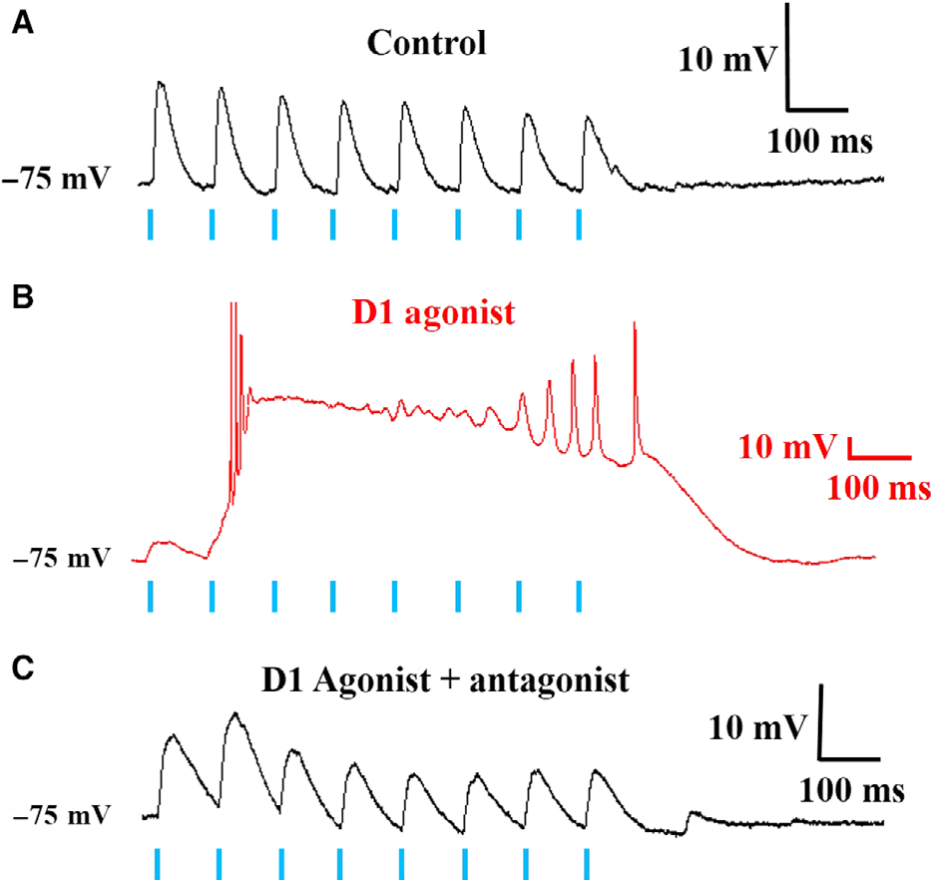
D1 receptor activation elicits bursting in a subset of PT pyramidal cells. (A) In control solution (gabazine containing), a train of eight EPSPs was elicited in PT cells by optogenetic activation of commissural fibers at 10 Hz. (B) Upon D1 receptor activation with bath applied SKF38393, the cell elicited a bursting pattern with the same stimulation protocol and light intensity. (C) In the presence of the D1 receptor antagonist (SCH23390), this bursting behavior was blocked. However, the time course of the EPSPs remained prolonged compared to control. Scale bars: *x* = 100 msec, *y* = 10 mV. Blue lines indicate 1ms blue LED light pulses.

In a subset of PT layer V pyramidal cells (3/9), application of SKF38393 in combination with commissural fiber stimulation elicited bursting behavior. Interestingly, cells that displayed bursting had an average decay time constant of 58.8 ± 15.0 msec, whereas nonbursting cells had an average decay time constant of 43.5 ± 2.8 msec (*P* > 0.05). Bursting evoked by a 10 Hz EPSP train is shown in Figure 7. Bursting was not observed in any IT cells under these conditions. Interestingly, the D1 antagonist prevented bursting, but did not completely restore EPSP responses to control within 5 min of application. Although SCH23390 prevented bursting and returned the initial EPSP amplitude to control levels (control: 5.2 ± 0.85 mV; D1 agonist + antagonist: 6.7 ± 2.9 mV), EPSPs remained prolonged (i.e. the EPSP decay time constant was longer than observed in control solution [control: 45.2 ± 2.9 msec; D1 agonist + antagonist: 51.5 ± 13.2 msec). This suggests that D1 receptor activation may enhance bursting capabilities in a subset of PT cells.

## Discussion

### Summary

In the present study, PT and IT layer V pyramidal neurons were distinguished based on their projection patterns and electrophysiological properties. Commissural inputs into PT and IT cells display facilitating and depressing patterns of EPSPs, respectively. D1 receptor activation increased the amplitude, and enhanced facilitation of commissural‐evoked EPSPs in PT cells, while D1 receptor activation increased the amplitude of commissural‐evoked EPSPs in IT cells. Finally, D1 receptor activation elicited burst firing in a subset of PT layer V pyramidal cells in response to activation of commissural fibers, unmasking a distinction between “regular spiking” and “burst firing” PT pyramidal cells.

### Comparisons with previous studies

Some studies have suggested that commissural activation onto PT and IT pyramidal cells results in different EPSP characteristics. As reported by Dembrow and colleagues (2015), we also observed that EPSPs evoked by optogenetic activation of commissural fibers onto layer V cells have a longer decay time constant in IT cells compared to PT cells. Additionally, as observed by Dembrow et al. (2015) we also report that the rise times of commissural‐evoked EPSPs elicited in PT and IT cells are not significantly different (Dembrow et al., 2015).

Also consistent with other studies (Lee et al., 2014), we report that PT and IT cells exhibit facilitating and depressing responses, respectively, when excited by repetitive commissural fiber stimulation. In addition to reports of differences in local connectivity (Wang et al., 2006; Brown and Hestrin, 2009; Morishima et al., 2011; Lee et al., 2014), Wang and colleagues (2006) suggest that some layer V pyramidal cells display only facilitating EPSP patterns, while others only display a depressing pattern. However, this study did not distinguish between layer V pyramidal subtypes. Interestingly, with paired dual recordings, Morishima and colleagues (2011) reported that IT inputs into PT cells displayed either facilitating or depressing responses. However, using optogenetic activation, Lee et al. (2014) reported that commissural activation of PT and IT cells results in facilitating and depressing paired‐pulse responses, respectively ( Lee et al., 2014).

Finally, in a previous study, we used local stimulation of layer V to characterize evoked EPSP trains in layer V pyramidal cells (Leyrer‐Jackson and Thomas, 2017; Leyrer‐Jackson and Thomas, 2018). In contrast to the present study, the majority of axonal fibers activated by direct stimulation of layer V is comprised of a mixture of PT and IT axons, and the evoked EPSPs showed a mixture of facilitating and depressing responses that was not correlated with postsynaptic cell type. In the current study, we selectively activated contralateral fibers, and we were able to attribute facilitating and depressing EPSP trains to postsynaptic PT and IT cells, respectively. Thus, our current results support the hypothesis that layer V pyramidal cell subtypes integrate synaptic inputs differently, and these results are consistent with previous studies that used a similar protocol (e.g. Lee et al., 2014).

Regarding the effects of D1 receptor activation, our current results are in line with results from our previous study (Leyrer‐Jackson and Thomas, 2018). As reported here, D1 receptor activation increased the amplitude of EPSPs evoked in both subtypes of layer V pyramidal cells by direct layer V stimulation. The similar effects of D1 receptor activation that we observed support the idea that the majority of commissural fibers is targeting layer V. Furthermore, Anastasiades et al. (2018) have also reported that D1 receptor activation enhances cortico‐cortical connectivity, supporting the results reported here. Contrary to results reported here, others have shown that D1 receptor activation suppresses commissural fiber‐evoked EPSCs in layer V pyramidal cells through a presynaptic mechanism involving modulation of calcium channels (Burke et al., 2018). However, these studies were conducted without the presence of GABA blockers, thus likely involving D1 modulatory effects on GABAergic interneurons. Furthermore, this study did not dissect differences in prefrontal cell type in which recordings were conducted. Thus, while importantly showing that D1 receptor activation can suppress specific inputs targeting the prefrontal cortex, these results are likely opposing due to differences in methodology.

### Locus of action of D1R effects

Regarding the locus of action of the D1R agonist on layer V pyramidal cell subtypes, there have been seemingly contrary results published. Seong and Carter showed specific D1R modulation of presumed IT layer V pyramidal cells (determined by lack of a voltage sag in response to hyperpolarizing current injection, not retrolabeling), suggesting that PT cells do not express D1Rs (Seong and Carter, 2012). However, Narayanan’s lab demonstrated (using CreloxP techniques) that a subset of D1R‐expressing cells in mPFC project to brainstem, hypothalamus, and other subcortical nuclei (Han et al., 2017); these are PT cells by definition. Additionally, Sohal’s lab has provided evidence that, while only PT cells might express D2Rs, both subtypes express D1Rs (Gee et al., 2012). Thus, it appears that there is a subset of PT cells that express D1Rs postsynaptically. In the current study, we observed opposing effects of D1R activation on synaptic dynamics (EPSP2/EPSP1 ratio) in recordings from PT cells; it is tempting to hypothesize that this may reflect these different populations of PT cells that do, or do not, express postsynaptic D1Rs. Future studies will be required to address this interesting possibility.

### Implications for prefrontal function

To our knowledge, this study is the first to characterize the effects of D1 receptor activation on optogenetically activated commissural inputs targeting PT and IT cells. However, it is known that PT cells are the only subtype of layer V cells that can fire persistently in response to neuromodulators (Dembrow et al., 2010), whereas IT cells primarily function as temporal integrators (Morishima et al., 2011; Dembrow et al., 2015). Our results suggest that D1 receptor activation could promote persistent firing in PT cells by increasing commissural‐evoked EPSP amplitude, promoting facilitation, and promote synaptic integration by widening the timing window of EPSPs. As this study is the first to identify the effects of dopamine on commissural inputs targeting layer V pyramidal cells, it is difficult to compare these results to other studies. However, one study suggests that moderate D1 receptor activation can promote persistent firing in deep prefrontal cortical neurons by producing prolonged depolarization in response to a train of inputs (Kroener et al., 2009); taken together with our data, these observations strongly support the hypothesis that dopaminergic activation of D1‐type receptors can promote persistent firing in PT cells.

Furthermore, our results suggest that D1 receptor activation may promote temporal integration of commissural inputs into IT cells by enhancing EPSP amplitude. Because these cells cannot be induced to fire persistently in the presence of neuromodulators (Dembrow et al., 2010), we hypothesize that D1 receptor activation may have a differential effect on commissural EPSP integration impinging onto IT cells. Interestingly, Anastasiades and colleagues (2018) have shown enhancement of cortico‐cortical connectivity through D1 receptor expressing pyramidal cells and interneurons. While not directly relevant to the current study, this study also suggests an enhancement in cortico‐cortical activity and therefore heightened communication between hemispheres, a phenomenon shown to play an important role in delay period activity (Li et al., 2016), essential for working memory tasks (Liu et al., 2014). Furthermore, while not a focus of the current study, Anastasiades and colleagues (2018) report that D1 receptor activation also activates vasoactive intestinal peptide expressing interneurons (VIP+) within the prefrontal cortex and promotes disinhibition of prefrontal microcircuits (Li et al., 2016). Others have shown that activation of VIP + interneurons inhibits somatostatin interneurons, promotes circuit excitation and enhances cognitive and behavioral function (Kamigaki and Dan, 2017; Garcia Del Molino et al., 2018). Our current results thus contribute to the hypothesis that optimal D1 receptor activation facilitates working memory by promoting recurrent excitatory synaptic activity between cortical regions.

Lastly, we report that dopaminergic D1 receptor activation can lead to burst firing in a subset of PT layer V pyramidal cells in response to commissural fiber activation. Thus, D1 receptor activation in combination with commissural fiber stimulation may uncover the subset of PT layer V pyramidal cells, identified previously as “intrinsic bursting” or IB cells (Yang et al., 1996). This is an intriguing finding, since it has been hypothesized that burst firing occurs with activation of both apical and basal dendritic compartments, concurrent with an apical calcium action potential (Larkum et al., 1999). While not significant, we report that bursting PT cells show longer EPSP decay time constants compared to nonbursting PT cells. Thus, D1 receptor activation may promote bursting in a subset of PT cells by enhancing NMDA receptor activation in basal dendrites (Leyrer‐Jackson and Thomas, 2018) and conceivably, NMDA spikes (Vincent et al., 1993). However, further exploration would be warranted to make definitive conclusions regarding the mechanism of D1R‐induced burst generation. Since many commissural fibers synapse on basal dendrites of IT and PT cells within layer V (Dembrow et al., 2015; Anastasiades et al., 2018), and D1 receptors are more abundant within the deep cortical layers (Gaspar et al., 1995; Scheler and Fellous, 2001; Anastasiades et al., 2018) our data suggest that D1 receptor activation may promote burst firing initiated by enhancing basal synaptic input. Whether this effect is limited to commissural fiber activation is currently unknown, and whether the effect has any physiological significance requires further studies.

## Conclusion

The data presented here suggest that D1 receptor activation may promote persistent firing and temporal integration in PT and IT cells, respectively. Dopamine can play an important role in reverberant and persistent neuronal activity within the mPFC (Scheler and Fellous, 2001), and it is known that proper levels of dopamine are essential for memory‐related tasks. However, it remains unclear where dopamine acts to modulate such functions. Thus, our data suggest that normal activation of D1 receptors may aid in working memory‐related tasks by enhancing persistent activity of PT cells and enhancing temporal integration of IT cells. Based on our results and those of others, it is conceivable that insufficient activation of D1 receptors in schizophrenic patients (i.e. during a depressive state) may lead to distractibility due to an inability to maintain recurrent activity between layer V output cells. In contrast, during hyperdopaminergic states (i.e. during psychotic episodes) excessive activation of D1 receptors may lead to perseveration due to inappropriate layer V cell firing. Also, the former would contribute to poor performance on working memory tasks, while the latter could contribute to false attributions regarding contextual information and the disorganized thought patterns commonly observed in the disorder.

## Conflict of Interest

None declared.
